# Does the introduction of pre‐operative cardiopulmonary exercise testing in radical cystectomy delay or alter surgical care?

**DOI:** 10.1002/bco2.133

**Published:** 2021-12-12

**Authors:** Kathleen R. Lockhart, Rosemary Carroll, Albert Tiu, Alison Blatt

**Affiliations:** ^1^ John Hunter Hospital Newcastle New South Wales Australia

**Keywords:** anaerobic threshold, bladder cancer, cardiopulmonary exercise test, perioperative care, radical cystectomy

## Abstract

**Objectives:**

To assess if the introduction of routine pre‐operative cardiopulmonary exercise testing (CPET) in radical cystectomy has delayed surgical intervention.

**Materials and Methods:**

A prospective database of patients undergoing radical cystectomy in our local health network was maintained. A retrospective analysis of two years (2018–2020) included 38 patients. Of these, 15 patients had CPET pre‐operatively, and a direct comparison was performed.

**Results:**

The mean time from diagnosis to cystectomy was 95 days in patients who did not have CPET compared to 110 days for those who did (*p* = 0.32), with comparable rates of neoadjuvant chemotherapy (NAC) (62.5% and 64.29%). Average length of stay was 18.6 days compared with 13.87 (*p* = 0.16), favouring the CPET group. The CPET group also had a lower readmission rate within 30 days (13.33% compared with 21.05%, *p* = 0.35). Cause‐specific mortality within 90 days was 10.2% and within the study timeframe was 36.84% (estimated 5‐year mortality rate 43–65%). Within the CPET group, eight had an anaerobic threshold (AT) of <11 ml/kg/min (range 6.3–10.5): Of these, 50% had Clavien‐Dindo complications of grade 2 or higher and the 90‐day mortality rate was 37.5% (cf. 0% in those with AT > 11 ml/kg/min in this series).

**Conclusion:**

CPET is a valuable risk evaluation tool. This study suggested that CPET contributed to a minor non‐significant delay to surgery, however was associated with reduced length of stay and readmission rates, and was a valuable risk evaluation tool. We found that CPET AT <11 ml/kg/min is associated with higher rates of patient morbidity and perioperative mortality.

## 1 INTRODUCTION

Radical cystectomies are performed for muscle invasive and high risk or recurrent non‐muscle invasive bladder cancer, a major operation with significant morbidity (significant complications in ~30%, overall complications up to 64%) and a perioperative 90 day mortality rate of 5–8%.^1^, ^2^ When adjusted for comorbidity and smoking status, age itself is not deemed a risk factor for post‐operative morbidity/mortality, rather cardiorespiratory fitness status.^1^, ^2^, ^3^, ^4^

CPET is a non‐invasive and reproducible method of assessing cardiorespiratory function and reserve through a closely monitored exercise test. Its focus is measuring oxygen uptake (VO_2_) (and calculating ventilatory equivalent for carbon dioxide [VE/VCO_2_]) during exercise and the point at which aerobic respiration is supplemented by anaerobic respiration to produce energy (the anaerobic threshold [AT]), which appears to correlate with peri‐operative outcomes.^2^, ^5^

CPET is being adopted increasingly into the pre‐operative workup and optimisation of patients prior to major elective non‐cardiac surgeries.^6^ It has played a role in Early Recovery After Surgery (ERAS) protocols for abdominal surgery and surgical p/rehabilitation.^4^, ^7^, ^8^ CPET can help risk assessment for perioperative morbidity and mortality (therefore guide perioperative care and anaesthetic practice), inform multidisciplinary decision‐making, assess optimisation of comorbidities, identify underlying pathology, and evaluate the effect of neoadjuvant therapies.^8^ This is particularly important in non‐cardiothoracic surgery such as cystectomy where there are increased cardiovascular demands without the expected cardiorespiratory improvement from the intervention itself.^9^ However, there is paucity of studies in the literature assessing the realistic impact of introducing CPET‐driven pre‐operative co‐morbid optimisation, or any service‐delivery related learning curve, on time to surgery.

Studies have suggested poor CPET scores pre‐operatively are correlated with a longer and more morbid length of stay.^2^, ^5^, ^10^ For radical cystectomy, post‐operative functional performance has also been found to correlate with overall survival.^3^, ^11^ Patients with a low AT of ≤11–12 ml/kg/min and VE/VCO_2_ ≥ 33 appear to be at higher risk of complications and mortality.^2^, ^5^ However, the literature is still relatively sparse for CPET in radical cystectomy (either laparoscopic/robotic or open) compared with other major abdominal surgery, and results have been to some degree divided. The best variables to predict outcome risks are still being clarified and validated.^2^, ^8^, ^12^

This pilot study aims to identify whether the introduction of CPET prior to radical cystectomy resulted in delay to surgical intervention. The secondary outcomes assessed included comparison of length of stay, readmission within 90 days, post‐operative morbidity and mortality rates.

## 2 MATERIALS AND METHODS

All patients aged 18 years and over who underwent open radical cystectomy at John Hunter Hospital from January 2018 to December 2020 were included. Pre‐operative CPET became routine for cystectomies in December 2019; CPET testing is performed on a calibrated electronically‐braked cycle ergometer, with patients wearing a 12‐lead ECG and being monitored by an exercise physiologist and medical doctor. The rate at which power increases each minute is individualised, with the aim of achieving a test duration of between 8 and 12 min. Breath‐by‐breath expired gas analysis is undertaken using a Medisoft Exp'air metabolic cart. Peak exercise capacity is defined as the rate of oxygen consumption averaged over the final 30 second epoch of the CPET. AT at our institution is determined using the Modified V‐slope method (in a plot of the minute production of CO_2_ [VCO_2_] over the minute uptake of oxygen [VO_2_], the slope increases from <1 to >1). If this is not possible, secondary methods (ventilatory equivalent method or excess carbon dioxide method) are utilised to determine AT.

The included patients were divided into two groups based on whether they had pre‐operative CPET assessment or not. The primary outcome of interest was whether the introduction of CPET resulted in delay to surgical intervention. Secondary outcomes included length of inpatient stay, perioperative, and postoperative morbidity/mortality outcomes as a result of CPET assessment and potential change in patient selection. Data obtained from the medical record included basic demographics, comorbidities expressed as a calculated Charlson comorbidity index (excluding their bladder cancer), pre‐operative haemoglobin and albumin, histopathology results and staging, time from histopathological diagnosis to cystectomy, details of pre‐operative CPET assessment, perioperative or postoperative complications, length of ICU and hospital stay, readmissions, and post‐operative survival.

Ethics approval was obtained from Hunter New England Human Research Ethics Committee, a NSW Health Lead HREC and informed consent obtained from participants.

Comparative analysis was completed including a Kaplan–Meier survival curve, with 95% confidence intervals where indicated and *p* < 0.05 considered statistically significant.

## 3 RESULTS

Thirty‐eight patients were included over the two‐year retrospective study period. Overall, the mean age at diagnosis was 64 years and patients waited an average of 100.8 days from diagnosis to cystectomy. Twenty‐three patients had neoadjuvant chemotherapy (60.5%), and an additional seven had adjuvant chemotherapy. The mean albumin pre‐operatively was 35.94 g/L, and mean drop in haemoglobin from pre‐operatively to immediately post‐operatively was 23.84 g/L. The mean hospital length of stay was 16.05 days, whilst average ICU length of stay was 0.14 days. The readmission rate within 30 days was 21.05%. The overall cause‐specific mortality within the study timeframe was 36.84%, and the 90 day mortality rate was 10.2% (four patients).

These patients were divided into Group 1 (no CPET assessment, *n* = 23) and Group 2 (CPET assessment, *n* = 15) for comparative analysis (Table 1). Group 1 had a shorter diagnosis to cystectomy interval, 95 days compared with 110 (*p* = 0.32). Notably, COVID 19 may have affected elective surgery bookings in 2020 so a separate analysis of the period between January 2018–February 2020 and March 2020–December 2020 was performed, resulting in a mean of 97.81 days and 108.7 days respectively, without statistical significance (*p* = 0.508).

**TABLE 1:** Comparison of baseline characteristics and main outcomes

	Overall (*n* = 38)	Group 1 (no CPET, *n* = 23)	Group 2 (CPET pre‐op, *n* = 15)	Difference
Age at diagnosis (mean)	64.66	65.87	62.8	95% CI −9.6 to 3.46, *P* = 0.3467
Charlson comorbidity index (mean)	3.26	3.21	2.86	95% CI −1.55 to 0.85, *P* = 0.5594
Difference in Hb pre‐operatively to post‐operatively (g/L, mean)	23.84	24.83	22.14	95% CI −13.62 to 8.24, *P* = 0.6208
Pre‐operative albumin (g/L, mean)	35.95	36.17	35.57	95% CI −3.86 to 2.66, *P* = 0.7108
Mean (days) from diagnosis prompting surgery to cystectomy date	100.83	95.34	110.54	95% CI −15.37 to 45.75, *P* = 0.3196
Hospital LOS (days, mean)	16.05	18.56	13.87	95% CI −11.28 to 1.88, *P* = 0.1563
ICU LOS during admission (days, mean)	0.14	0.48	0.2	95% CI −22.60 to 26.01, *P* = 0.7406
Readmission within 30 days	8/38 = 21.05%	6/23 = 26.08%	2/15 = 13.33%	95% CI −15.28 to 35.27, *P* = 0.3524
For those who died within study period				
Mean post operative survival time (days) (percentage mortality)	255.2143 (*n* 14/38 = 36.84%)	306 (*n* 9/23 = 39.13%)	163.8 (*n* 5/15 = 33.33%)	95% CI −271.67 to −12.72, *P* = 0.0323
Median survival (days)	180.5	183	82	
Interquartile range (IQR) (days)	80.75–388	152.5–457.5	68.5–300	
90‐day mortality (*n*, percentage)	4/39 = 10.2%	1/23 = 4.35%	3/15 = 20.0%	95% CI −5.44 to 41.09, *P* = 0.1295

Both groups had comparable baseline characteristics and comparable rates of neoadjuvant chemotherapy (NAC) (62.5% and 64.29%). Average length of stay was 18.6 days compared with 13.87 (*p* = 0.16), favouring Group 2. The CPET group also had a lower readmission rate within 30 days (13.33% compared with 21.05%, *p* = 0.35).

**FIGURE 1:**
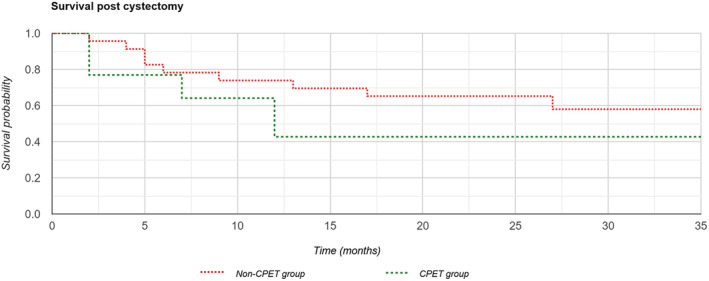
Kaplan–Meier survival curve

**FIGURE 2:**
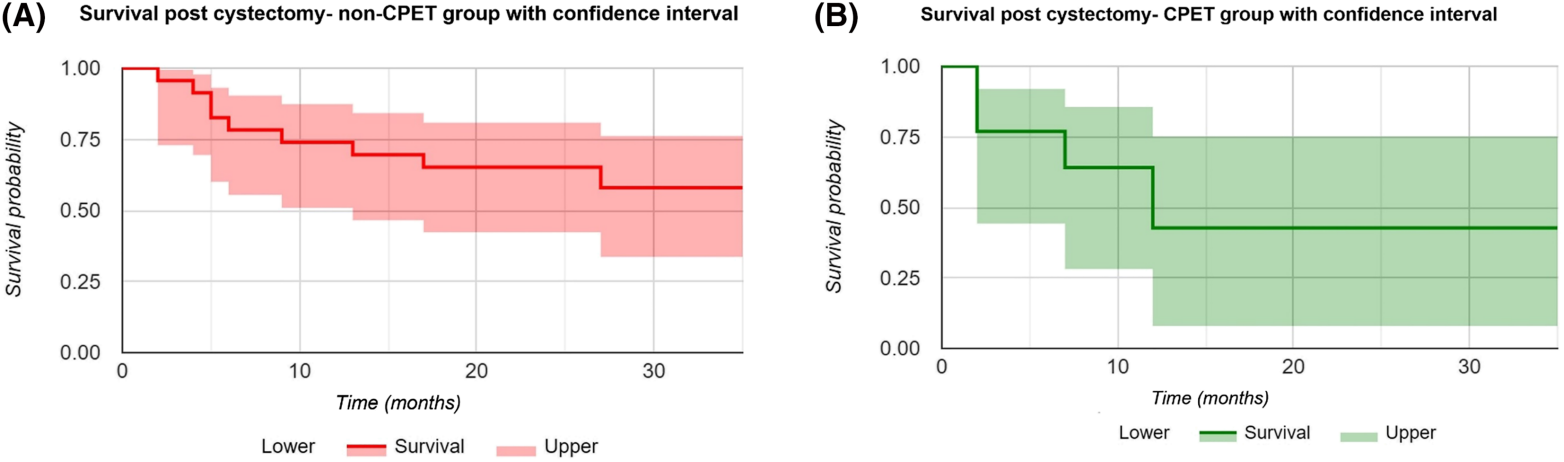
(A) Kaplan–Meier survival curve for Group 1 (no CPET) with confidence interval; (B) Kaplan–Meier survival curve for Group 2 (pre‐operative CPET) with confidence interval

A Kaplan–Meier analysis was completed which demonstrated a survival rate of 65% (Group 1) compared with 43% (Group 2) at 24 months, but this was not statistically significant (*p* = 0.205) (Figures 1 and 2).

Within the CPET group, the mean peak VO_2_ was 18.49, mean AT was 11.89, and VE/VOC_2_ was 35.77. CPET values correlated with poor outcomes, particularly AT, which has also been favoured in the literature as a predictor of post‐operative morbidity. In this study, AT of <11 ml/kg/min was associated with an increased rate of post‐operative complications. Eight patients had an AT <11 ml/kg/min (range 6.3–10.5): Of these patients, 50% had Clavien–Dindo complications of grade 2 or higher, and the 90‐day mortality rate was 37.5% (this is compared to 0% in those with AT>11 ml/kg/min in Group 2). Of the five patients who had the lowest pre‐operative AT measurements at <9 (range 6.3–8.8), four had a Clavien–Dindo complication of II or higher (80%).

## 4 DISCUSSION

We have established in this study that the introduction of CPET itself did not cause significant delays to surgery in our local health district. Patients who had CPET appeared to have a shorter length of stay and lower readmission rate. However, this change in hospital stay outcomes suggests that although the demographics of the two groups were comparable, the process of having CPET may result in some degree of selection bias; the scope of this study did not include all potential candidates considered for cystectomy, only those who proceeded to surgery. In the study period, routine CPET was still being established and prehabilitation had not yet been implemented as a formal program, although it has now been introduced to optimise high risk patients. The beneficial difference then, noting that it is not statistically significant, may be attributable to a small cohort and other confounders.

In reviewing the patients who had <90‐day mortality, all were cause‐specific (metastatic bladder cancer), and all had either aggressive undifferentiated or variant pathology or were upstaged at cystectomy to at least nodal metastatic disease. This likely accounts for the slightly higher than average proportion of early mortality, 10.2% compared with ~8% quoted in the literature.^13^ However, some studies have suggested that the mortality rate post cystectomy is likely to have been previously under‐reported in the literature. Schiffmann et al.'s study used data from a SEER‐medicare database from 1991 to 2009 and reported an average 90‐day mortality rate of 10.6% (with statistically significant predictors of increased mortality including: advanced age, higher CCI, low socioeconomic status, unmarried status and non‐organ confined staging).^14^ Continuation of this study with risk stratifying patients according to pathology would provide more informative subgroup analysis with a larger cohort. The 5‐year cancer‐specific survival rate post cystectomy is between ~25–63% in the literature.^13^, ^15^ Our 24‐ to 35‐month survival rate calculated is also comparable to other studies (52–71%), but further longer term studies and specific subgroup analysis with tumour staging, grading, and variant pathology would be beneficial in considering the impact of CPET and prehabilitation options on outcomes.^16^, ^17^, ^18^

Anaerobic threshold is considered the optimal predictor of outcome in intra‐abdominal surgery, with <10–11 ml/kg/min considered high risk.^2^, ^5^, ^19^ We certainly found this to be consistent with our post‐operative morbidity and mortality outcomes showing higher complication rates and mortality rates with patients who had AT <11 ml/kg/min.

Given the small number of patients who had CPET done, subgroup analysis beyond overall morbidity/mortality rates was not useful; however, larger studies evaluating the survival statistics of patients with stratified AT results would be useful in further assessment of whether CPET and pre‐operative optimisation programs impacts this.

Limitations of this study include its retrospective design and limited cohort size which introduce potential for bias; additional studies would be beneficial with an extended time frame including prospective data collection and a longer follow up period. During the course of this study, the effect of COVID 19 on elective surgery and pre‐operative assessment may have been a confounder in delaying time to surgery.

CPET is a valuable risk evaluation tool. This study suggested that CPET contributed to a clinically non‐significant delay to surgery and may result in reduced length of stay and readmission rates. We found CPET AT <11 ml/kg/min is associated with higher rates of patient morbidity and perioperative mortality.

## CONFLICT OF INTEREST

All authors declare that they have no conflict of interest or additional sources of support or funding to declare.

## CONSENT FOR PUBLICATION

All included data were de‐identified, and no direct patient participation was required, but consent will be sought with future prospective data collection for further study.

## ETHICS APPROVAL AND CONSENT TO PARTICIPATE

This research study was conducted retrospectively from data obtained for clinical purposes. It was registered and approved (identifier 2020/STE01913) with the research ethics and governance information system (REGIS).

## GUARANTOR

AB

## AUTHOR CONTRIBUTIONS

KL and AB conceived the study. KL researched the literature, developed the protocol, and obtained ethics approval with RC. KL drafted the manuscript and performed data analysis. AT and AB supervised the study, and AB edited and approved the final manuscript. All authors have read and approved the manuscript.

## Data Availability

The data analyses generated are available within the presented study but specific datasets are kept confidential due to their nature of including clinical and radiological details. Further information regarding dataset analysis may be available from the corresponding author upon request.

## References

[1] Young MJ , Elmussareh M , Weston P , Dooldeniya M . Radical cystectomy in the elderly—is this a safe treatment option? Arab J Urol. 2017 Oct 5;15(4):360–5. 10.1016/j.aju.2017.09.002 29234541 PMC5717452

[2] Tolchard S , Angell J , Pyke M , Lewis S , Dodds N , Darweish A , et al. Cardiopulmonary reserve as determined by cardiopulmonary exercise stress testing correlates with length of stay and predicts complications after radical cystectomy. BJU Int. 2015;115:554–61. 25109512 10.1111/bju.12895

[3] Porserud A , Karlsson P , Rydwik E , Aly M , Henningsohn L , Nygren‐Bonnier E , et al. The CanMoRe trial—evaluating the effects of an exercise intervention after robotic‐assisted radical cystectomy for urinary bladder cancer: the study protocol of a randomised controlled trial. BMC Cancer. 2020 Aug 26;20(1):805. 10.1186/s12885-020-07140-5 32842975 PMC7448437

[4] Levett DZ , Grocott MP . Cardiopulmonary exercise testing, prehabilitation, and Enhanced Recovery After Surgery (ERAS). Can J Anaesth. 2015 Feb;62(2):131–42. 10.1007/s12630-014-0307-6 Epub 2015 Jan 22 25608638 PMC4315486

[5] Prentis JM , Trenell MI , Vasdev N , French R , Dines G , Thorpe A , et al. Impaired cardiopulmonary reserve in an elderly population is related to postoperative morbidity and length of hospital stay after radical cystectomy. BJU Int. 2013;112(2):E13–9. 23795790 10.1111/bju.12219

[6] McGrath JS . Cardiopulmonary exercise testing: fortune‐teller or guardian angel? BJUI. 2015;4(115):502–3. 10.1111/bju.12962 25808710

[7] Pang KH , Groves R , Venugopal S , Noon AP , Catto JWF . Prospective implementation of enhanced recovery after surgery protocols to radical cystectomy. Eur Urol. 2018 Mar;73(3):363–71. 10.1016/j.eururo.2017.07.031 Epub 2017 Aug 8 28801130

[8] Levett DZH , Jack S , Swart M , Carlisle J , Wilson J , Snowden C , et al. Perioperative Exercise Testing and Training Society (POETTS). Perioperative cardiopulmonary exercise testing (CPET): consensus clinical guidelines on indications, organisation, conduct and physiological interpretation. Br J Anaesth. 2018 Mar;120(3):484–500. 10.1016/j.bja.2017.10.020 Epub 2017 Nov 24 29452805

[9] Khan M , Amoroso P , Gulati S . Cardiopulmonary exercise testing in major urological surgery: an old test; a new perspective; a potential application. BJU Int. 2013 Aug;112(4):E232–3. 10.1111/bju.12352 23879908

[10] Smith TB , Stonell C , Purkayastha S , Paraskevas P . Cardiopulmonary exercise testing as a risk assessment method in non cardio‐pulmonary surgery: a systematic review. Anaesthesia. 2009;64:883–93. 19604193 10.1111/j.1365-2044.2009.05983.x

[11] Wilson RJ , Davies S , Yates D , Redman J , Stone M . Impaired functional capacity is associated with all‐cause mortality after major elective intra‐abdominal surgery. Br J Anaesth. 2010 Sep;105(3):297–303. 10.1093/bja/aeq128 Epub 2010 Jun 23 20573634

[12] Older PO , Levett DZH . Cardiopulmonary exercise testing and surgery. Ann Am Thorac Soc. 2017;S74–83. 10.1513/AnnalsATS.201610-780FR 28511024

[13] May M , Helke C , Nitzke T , Vogler H , Hoschke B . Survival rates after radical cystectomy according to tumor stage of bladder carcinoma at first presentation. Urol Int. 2004;72(2):103–11. 10.1159/000075962 14963349

[14] Schiffmann J , Gandaglia G , Larcher A , Sun M , Tian Z , Shariat SF , et al. Contemporary 90‐day mortality rates after radical cystectomy in the elderly. Eur J Surg Oncol. 2014 Dec;40(12):1738–45. 10.1016/j.ejso.2014.10.004 Epub 2014 Oct 15 25454826

[15] Cai Z , Jin H , Chen J , Hu J , Li H , Yi Z , et al. Adjuvant chemotherapy in patients with locally advanced bladder cancer after neoadjuvant chemotherapy and radical cystectomy: a systematic review and pooled analysis. Transl Androl Urol. 2021 Jan;10(1):283–91. 10.21037/tau-20-571 33532317 PMC7844510

[16] Lin HY , Ye H , Kernen KM , Hafron JM , Krauss DJ . National Cancer Database comparison of radical cystectomy vs chemoradiotherapy for muscle‐invasive bladder cancer: Implications of using clinical vs pathologic staging. Cancer Med. 2018 Nov;7(11):5370–81. 10.1002/cam4.1684 Epub 2018 Oct 10 30306728 PMC6247074

[17] Coughlin GD , Youl PH , Philpot S , Wright MJ , Honore M , Theile DE , et al. Outcomes following radical cystectomy: a population‐based study from Queensland, Australia. ANZ J Surg. 2019 Jun;89(6):752–7. 10.1111/ans.15259 Epub 2019 May 14 31087817

[18] Williams SB , Huo J , Chu Y , Baillargeon JG , Daskivich T , Kuo Y‐F , et al. Cancer and all‐cause mortality in bladder cancer patients undergoing radical cystectomy: Development and validation of a nomogram for treatment decision‐making. Urology. 2017 Dec;110:76–83. 10.1016/j.urology.2017.08.024 Epub 2017 Aug 25 28847688 PMC8336075

[19] Moran J , Wilson F , Guinan E , McCormick P , Hussey J , Moriarty J . Role of cardiopulmonary exercise testing as a risk‐assessment method in patients undergoing intra‐abdominal surgery: A systematic review. Br J Anaesth. 2016 Feb;116(2):177–91. 10.1093/bja/aev454 26787788

